# Direct measurement of fluorocarbon radicals in the thermal destruction of perfluorohexanoic acid using photoionization mass spectrometry

**DOI:** 10.1126/sciadv.adt3363

**Published:** 2025-02-28

**Authors:** Ming-Gao Xu, Chen Huang, Long Zhao, Anthony K. Rappé, Eric M. Kennedy, Michael Stockenhuber, John C. Mackie, Nathan H. Weber, John A. Lucas, Musahid Ahmed, Jens Blotevogel, Wenchao Lu

**Affiliations:** ^1^ National Synchrotron Radiation Laboratory, University of Science and Technology of China, Hefei, Anhui 230029, China.; ^2^ School of Nuclear Science and Technology, University of Science and Technology of China, Hefei, Anhui 230027, China.; ^3^ Department of Chemistry, Colorado State University, Fort Collins, CO 80523, USA.; ^4^ Discipline of Chemical Engineering, School of Engineering, University of Newcastle, Callaghan, New South Wales 2308, Australia.; ^5^ Oak Ridge Institute for Science and Education, Office of Research and Development, US Environmental Protection Agency, Research Triangle Park, NC 27711, USA.; ^6^ Department of Chemical and Biological Engineering, Monash University, Clayton, Victoria 3800, Australia.; ^7^ Veolia Environmental Services, Australia & New Zealand, Southbank, Victoria 3006, Australia.; ^8^ Chemical Sciences Division, Lawrence Berkeley National Laboratory, Berkeley, CA 94720, USA.; ^9^ CSIRO Environment, Waite Campus, Urrbrae, South Australia 5064, Australia.

## Abstract

Thermal destruction is a critical cornerstone of addressing the rampant contamination of natural resources with per- and polyfluoroalkyl substances (PFAS). However, grave concerns associated with stack emissions from incineration exist because mechanistic studies have thus far relied on ex situ analyses of end products and theoretical calculations. Here, we used synchrotron-based vacuum ultraviolet photoionization mass spectrometry to study the pyrolysis of a representative PFAS—perfluorohexanoic acid—and provide direct evidence of fluorocarbon radicals and intermediates. A key reaction pathway from perfluorocarboxylic acids to ketenes via acyl fluorides is proposed. We furthermore propose CF_2_/CF_3_ radical–centered pyrolysis mechanisms and explain their roles in the formation of other products that may form in full-scale incinerators. These results have not only unveiled the role of radicals and intermediates in thermal PFAS decomposition and recombination mechanisms but also provide unique insight into improving the safety and viability of industrial PFAS incineration.

## Teaser

Transient intermediates were identified during the incineration of PFAS, elucidating how incineration will work to destroy PFAS.

## INTRODUCTION

The widespread use of per- and polyfluoroalkyl substances (PFAS) over the last few decades has caused increasing concern due to their chemical stability, adverse human health, and ecosystem impacts. To manage PFAS in the environment, extensive research efforts have been made in examining their speciation (*1*), transport (*2*), adsorption (*3*), bioaccumulation (*4*), and toxicity (*5*). Meanwhile, there is an emergent development of technologies for PFAS destruction (*6*). Albeit entitled as “forever chemicals,” PFAS can be destroyed in both thermal-based (*7*) and non–thermal-based technologies including electrochemical oxidation, low-temperature plasma treatment, and sonolysis (*8*). However, thermal destruction is currently the only option for full-scale commercial treatment of large PFAS-affected waste streams, which applies to both PFAS-based materials (e.g., polytetrafluoroethylene) and PFAS-affected matrices [e.g., contaminated environmental media (*9*)].

In a typical hazardous waste industrial incinerator, organic chemicals are initially desorbed from the waste matrix in a primary combustion chamber such as a rotary kiln. In the secondary combustion chamber, also known as afterburner, the vaporized chemicals experience high temperatures ≥980°C (*10*) within 2 to 4 s of gas residence time, usually with the goal of ≥99.99% destruction (*11*). To understand the gas-phase incinerability of PFAS, a growing number of studies in recent years have investigated their thermal decomposition. The two most common PFAS subclasses found in the environment are perfluoroalkyl carboxylic acids (PFCAs) and perfluoroalkyl sulfonic acids (PFSAs). In previous studies, the thermal destruction of PFCAs and PFSAs was studied using Fourier transform infrared spectroscopy (FTIR) and gas chromatography–mass spectrometry (GC-MS) interfaced to pyrolytic apparatuses. For example, Xiao and coworkers used pyrolysis GC-MS to study thermal decomposition products of common PFAS in fluorinated aqueous film-forming foams (*12*–*14*) and in other media such as granular activated carbon (GAC) (*15*, *16*) and soils (*17*). Scission of the perfluorocarbon backbone was proposed as the initial decomposition steps leading to fluorocarbon radicals. Wang *et al.* (*18*) investigated the gas-phase products of PFCAs using a furnace coupled to FTIR spectroscopy, where fluorocarbon radicals were proposed as key intermediates, triggered by OH• radical oxidation. Weber *et al.* (*19*–*22*) constructed a laboratory-scale incinerator replicating industrial incineration coupled to an FTIR spectrometer for PFAS decomposition product distributions and kinetic studies. The authors also proposed that difluorocarbene (CF_2_) is a key product from perfluorocarbon backbone rupture at high temperatures, which may subsequently react with molecular oxygen or OH• via FCO• and COF_2_ to complete the mineralization of PFAS during incineration. Other techniques such as thermal desorption–pyrolysis–direct analysis in real time–mass spectrometry (*23*) were used to investigate the pyrolytic fragments as an in situ approach. However, direct evidence of potential thermal decomposition intermediates such as CF_2_, COF, and C*_n_*F_2*n*+1_, is still lacking because these radicals and intermediates are extremely reactive (e.g., the half-life of CF_2_ is 20 ms in the gas phase and 0.5 ms in solution) (*24*) and are challenging to detect by conventional approaches; yet, they are pivotal in understanding and hence forecasting thermal decomposition and recombination mechanisms.

The thermal decomposition mechanisms of PFCAs were recently revisited and summarized in fig. S1 based on theoretical work driven by ex situ experimental results (*11*, *25*–*28*). The kinetically most favorable reaction pathway is elimination of HF at the α-carbon (“α-HF elimination”), forming an α-lactone intermediate. At operating temperatures below 1200°C, α-HF elimination dominates over β C─C cleavage, releasing CO and leading to the formation of one-carbon-fewer perfluoroalkyl acyl fluorides (R_f_-COF). Alternatively, releasing CO_2_ will proceed at higher temperatures due to a higher free energy of activation (Δ^‡^*G*), forming perfluoro-1-alkenes and 1*H*-perfluoroalkanes. Previous calculations of perfluorooctanoic acid (PFOA) decomposition suggest that the corresponding Δ^‡^*G* are 29.3 kcal/mol for CO_2_ elimination and 14.4 kcal/mol for CO elimination, and counterions may indeed change the major mechanistic pathways (*11*). The successive thermal decomposition of R_f_-COF was proposed to cleave the β C─C bond and release the •CF_2_COF headgroup (*26*). The proposed mechanism via this route, however, lacks experimental validation especially because R_f_-COF species have not been characterized well but within in a few FTIR studies (*25*). In the presence of water, acyl fluorides hydrolyze rapidly (*26*); consequently, gas-phase hydrolysis of acyl fluorides in the water vapor–rich environment of an afterburner has been previously hypothesized (*29*). Recent experimental studies by Weber *et al.* (*21*, *22*) have confirmed that the addition of water vapor boosted the mineralization of perfluorooctane sulfonic acid (PFOS). At temperatures >850°C, a feed of 200 to 400 parts per million by volume (ppmv) of PFOS with 20,000 ppmv of water vapor in air produced only HF, SO_2_, and CO_2_ without gaseous fluorocarbons being detected (*22*). Theoretical calculations also suggested that R_f_-COF may hydrolyze into a PFCA by reacting with water vapor present in the system (*27*), followed by the successive elimination of CO and HF. This iteration shortens the carbon chain stepwise and leads to CO and HF being detected as final products. However, discrepancies in experimental results suggest that this route is preferred at lower temperatures compared to CO_2_ elimination, and shorter-chain PFCA intermediates were not detected except on GAC matrices (*30*). There is clearly a lack of direct experimental evidence for transient intermediates and radicals, leading to ambiguity in the proposed pathways to account for the observed experimental results (*25*). Moreover, the complexity and diversity of the reactor systems used by different research groups may introduce different secondary reactions (and/or surface reactions) between fluorocarbon intermediates and the furnace (*18*, *19*) and further complicate any mechanistic studies.

Molecular-beam mass spectrometry (MBMS), the method used in this work, operates by expanding higher-pressure gas-phase reactants into vacuum through a nozzle to form a supersonic jet. Therefore, MBMS permits nascent species with a very short residence time (microseconds to milliseconds) and a longer lifetime due to low molecular density and temperature (*31*, *32*), thus making them detectable after expansion. In recent decades, MBMS has been functionalized by tunable vacuum ultraviolet (VUV) photons from synchrotron facilities and versatile pyrolytic reactors to simulate real combustion and incineration processes. This has led to groundbreaking research to explore the combustion processes of hydrocarbons, such as thermal decomposition, oxidation, growth of polycyclic aromatic hydrocarbons, etc., which has successfully identified radicals, discovered previously unreported reaction mechanisms, and substantially expanded the mechanism framework (*31*, *33*). In the current study, we performed the pyrolysis experiments of a representative PFAS—perfluorohexanoic acid (PFHxA)—with and without water vapor, using synchrotron VUV photoionization mass spectrometry (SVUV-PIMS). We not only aim to provide a decisive molecular-level understanding of the pyrolysis mechanism of PFHxA but also adapt state-of-the-art technologies from molecular reaction dynamics to resolve outstanding issues in environmental chemistry as related to PFAS in the environment.

## RESULTS

### Detection of pyrolysis products

**Fig. 1.**
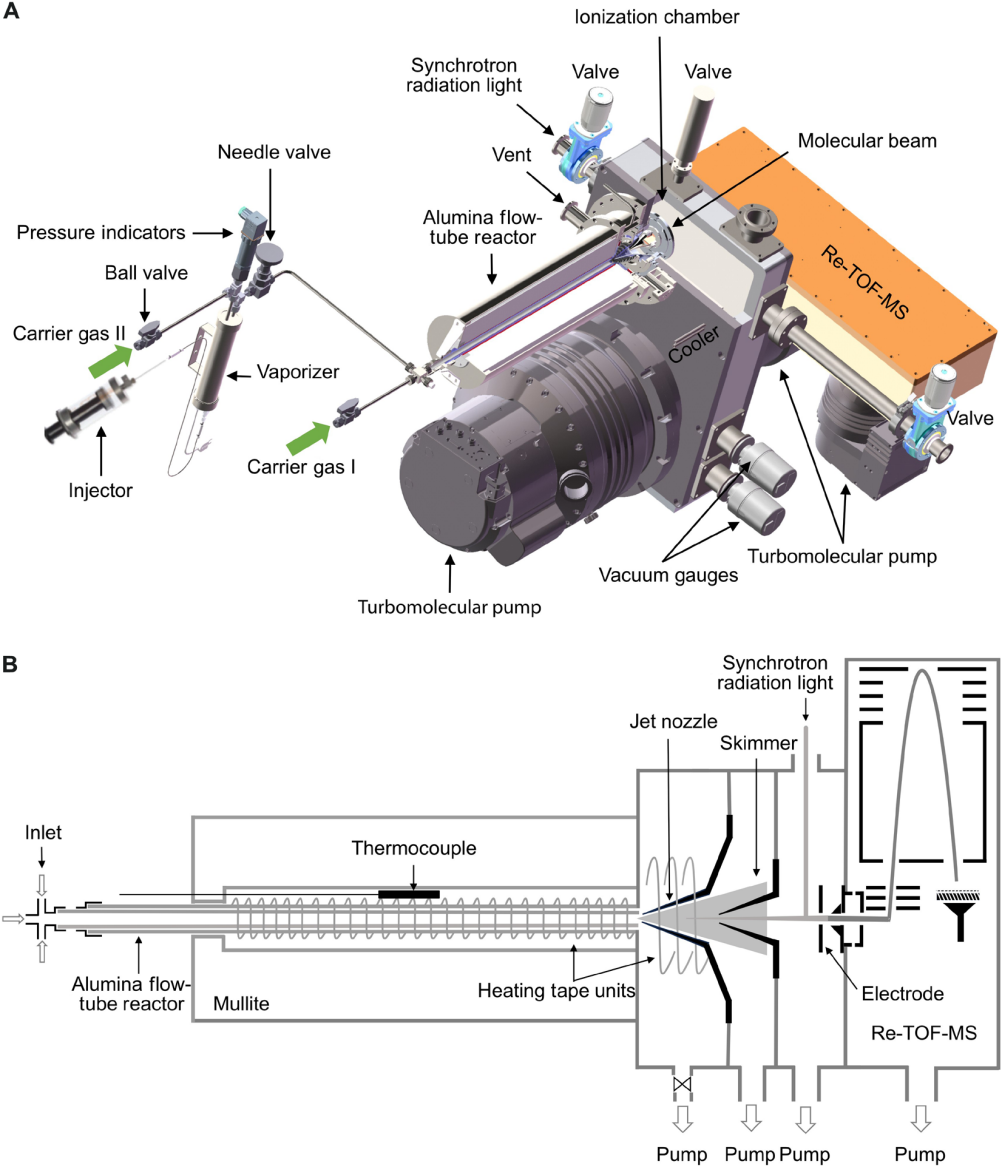
Structure of the synchrotron VUV photoionization mass spectrometer of this study. (**A**) Schematic view of the apparatus consisting of the injection system, the flow-tube reactor for pyrolysis, the ionization chamber, the TOF-MS chamber for detection, and the vacuum system, and (**B**) the cross section of the main body of the apparatus, showing the molecular beam path traversing the flow-tube reactor and nozzles, being ionized by synchrotron VUV photons and detected by the TOF-MS chamber.

We commence our investigation with the detection of pyrolysis products followed by their characterization and lastly our proposed mechanism. The design of the experimental setup is shown in Fig. 1 with more details discussed in Materials and Methods. The entire analytical detection process consists of four main steps: vaporization of the reactant, thermal decomposition, photoionization (PI), and reflectron time-of-flight mass spectrometry (Re-TOF-MS) analysis. During operation, 0.3% PFHxA vapor was seeded in argon carrier gas, passing through an alumina laminar flow tube with a temperature range from 400 to 975°C. The residence time is calculated to be around 10 ms as molecules traverse through the flow tube, where the high temperature caused thermal decomposition of PFHxA molecules. Other parameters of this reactor including its temperature profile, pressure, molecular density, and velocity have been well documented previously (*34*, *35*). Successively, the molecules expanded into vacuum to generate a supersonic molecular beam. The beam containing all thermal decomposition intermediates and products then entered the interaction region and was intersected by VUV photons from the synchrotron. When the photon energy exceeds the ionization energy (IE) of species A, PI will occur, resulting in a positively charged ion A^+^ by removal of an electron. Following ionization, all ionized species were then analyzed by the Re-TOF-MS component of the instrument. Because each molecule has its unique IE, it can be used for species identification when combined with mass-to-charge ratio (*m*/*z*) analysis. For example, when observing a mass spectral peak at *m*/*z* = 49.99, we could confirm it to be CF_2_^+^ only if this peak appeared at photon energies above 11.4 eV (the IE of CF_2_). When the photon energy was below 11.4 eV, CF_2_ remained neutral and could not be detected by the TOF-MS. Mass spectra were recorded at photon energies from 11.0 to 14.7 eV with increments of 0.5 eV. Photon energy at 18.0 eV was also scanned to detect species of high IEs, such as HF (IE = 16.00 eV), F (17.42 eV), F_2_ (15.69 eV), etc. (*36*). Temperatures higher than 1000°C were not considered because high temperature not only destroys the metastable species but also facilitates the reaction between free fluorine radicals and the flow tube reactor.

**Fig. 2.**
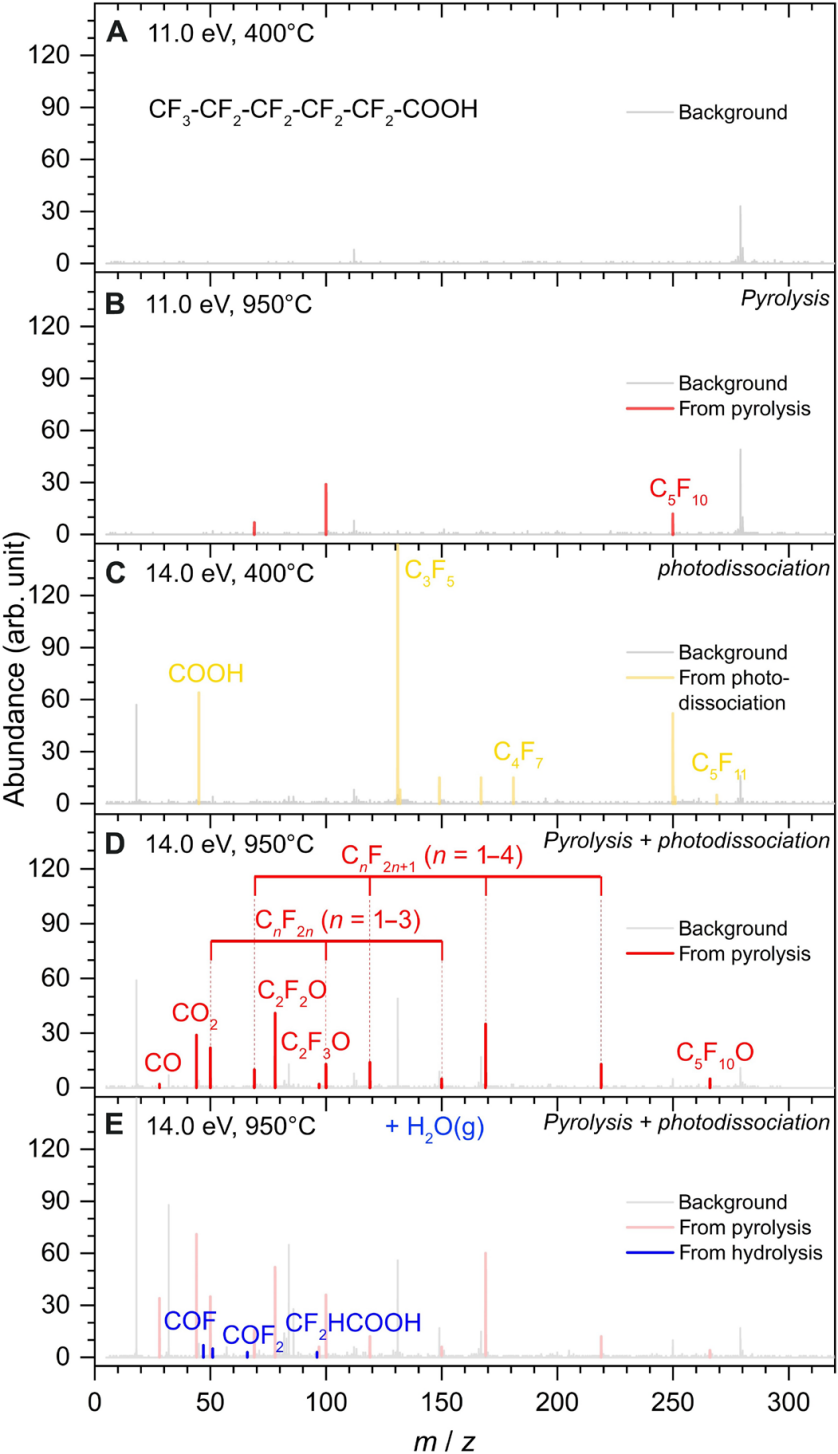
Mass spectra for the pyrolysis of PFHxA. (**A**) Mass spectrum collected at 11.0 eV and 400°C showing the background of the system. (**B**) Mass spectrum at 11.0 eV and 950°C showing the thermal decomposition products marked in red. (**C**) Mass spectrum at 14.0 eV and 400°C showing the photodissociation fragments of reactant PFHxA, marked in yellow. (**D**) Mass spectrum at 14.0 eV and 950°C showing all thermal decomposition products marked in red against background in gray. (**E**) Mass spectrum at 14.0 eV and 950°C with the addition of equal amount of water vapor to PFHxA. Additional species are marked in blue, in comparison with pyrolysis products (pale red peaks) and background (gray peaks). All identifiable pyrolysis products were labeled and characterized by *m*/*z* and ionization energies.

Beyond PI, VUV photons can induce molecular fragmentation through photodissociation (termed dissociative PI). To distinguish between genuine pyrolysis products and photodissociation fragments, we conducted a systematic investigation of the interplay between temperature and photon energy, as illustrated in Fig. 2. At low photon energy (11.0 eV) and low temperature (400°C), both parameters are below the threshold required for observing products. The only discernable peaks under these conditions are attributed to electronic noise or trace impurities in the sample. Upon increasing the temperature to 950°C, several fluorocarbon products and intermediates from pyrolysis emerged, while other pyrolysis products with higher IEs did not ionize and could not be detected at 11.0 eV. At 14.0 eV, most products were detected. High-energy photons could also cause dissociative PI fragments, which were identified and subtracted from matching pyrolysis product signals, such as C_5_F_11_• and •COOH radicals produced through photon-induced cleavage of the α C─C bond of PFHxA. Background gas remnants from the atmosphere such as O_2_ (IE = 12.07 eV) and H_2_O (IE = 12.62 eV) (*36*) were also accounted for. It is worth noting that certain species may be generated via both photodissociation of the reactant and pyrolysis processes. For example, C_5_F_10_ was observed as a pyrolysis product above 750°C with its IE = 10.6 eV. However, at 400°C and 11.5 eV, photodissociation of the parent PFHxA^+^ also generated C_5_F_10_ and other fragments simultaneously.

### Product characterization and temperature dependence

All detected pyrolysis products are summarized in table S1 together with their PI efficiency (PIE) curves in fig. S2. Thermal decomposition products started to emerge at 700°C in our experiments. Figure 2D shows the mass spectra collected at 950°C and the photon energy of 14.0 eV. High temperature enhanced the rupture of the perfluorocarbon backbones as predicted, forming a series of fluorocarbon radicals C*_n_*F_2*n*+1_• (*n* = 1 to 4) and difluorocarbene/perfluoroalkenes C*_n_*F_2*n*_ (*n* = 1 to 3, 5). CO_2_ and C_5_F_10_ were observed as decarboxylation products. CO and C_5_F_10_O (*m*/*z* = 265.98) appeared at the same temperature threshold (700°C), indicating that C_5_F_10_O and CO are from the same reaction pathway. Previous theoretical calculations (*11*, *25*–*28*) have suggested that perfluoropentanoyl fluoride (C_4_F_9_COF) is the decarbonylation product from PFHxA. This is the first direct mass spectrometric observation of acyl fluoride R_f_-COF from CO elimination of PFCAs. It is worth noting that an exotic intermediate C_2_F_2_O (*m*/*z* = 77.99 and IE = 12.2 eV) was observed. Previous theoretical work (*37*) has explored many possible isomers of C_2_F_2_O, with difluoroketene (CF_2_═C═O) being the stable global minimum structure. Experiments have reported evidence for the formation of CF_2_═C═O via the decomposition of bromodifluoroacetyl chloride/bromide (*38*) or via electron impact of a perfluoro methyl vinyl ether (*39*). •C_2_F_3_O (*m*/*z* = 96.99 and IE = 9.9 eV) is attributed to •CF_2_COF, the precursor of CF_2_═C═O, confirmed by the calculated IE at 9.94 eV. A highly unsaturated fluorocarbon, perfluoroallene (CF_2_═C═CF_2_, IE = 10.8 eV), was observed at >900°C. We did not detect the parent ion (PFHxA^+^, *m*/*z* = 313.98) over a range of photon energies possibly due to a high number of electronegative F atoms in the molecule. This photon-induced instability of PFHxA^+^ is reminiscent of the photolysis process of PFAS reported previously (*40*). A primary objective of PFAS pyrolysis is the conversion of fluorine atoms into HF. Under the conditions of this experiment, we were unable to detect HF up to 20.0 eV (see fig. S3, the mass spectrum collected at 950°C, 18.0 eV), possibly because of the low signal intensity and its reactivity with or absorption/retardation by the alumina reactor (*19*, *29*, *41*). For similar reasons, the F• radical, F_2_, and their hydration intermediates may have also been absent in the spectra.

**Fig. 3.**
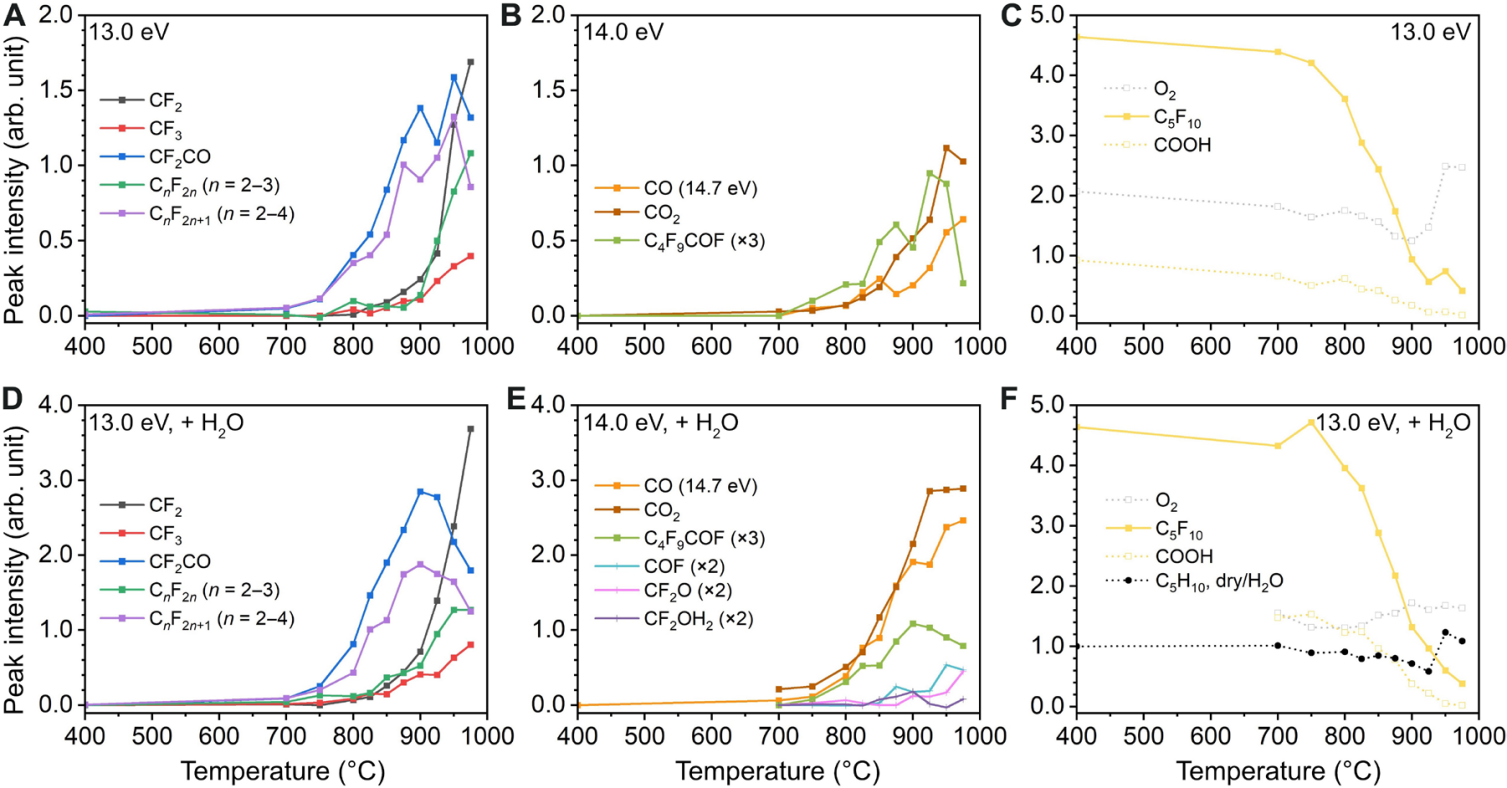
Temperature dependence curves for each pyrolysis product and some background species. (**A**) Temperature dependence curves (temperature versus integrated peak intensity) for fluorocarbon-based products [CF_2_, CF_3_•, CF_2_CO, C*_n_*F_2*n*_ (*n* = 2 to 3), and C*_n_*F_2*n*+1_• (*n* = 2 to 4)] at 13.0 eV, and (**B**) carbon oxides and C_4_F_9_COF at 14.0 eV (CO at 14.7 eV). Background gases (O_2_) and photodissociation products (C_5_F_10_ and •COOH) are shown in (**C**). (**D**) Temperature dependence curves with the addition of equal amount of water vapor to PFHxA for fluorocarbon-based products [CF_2_, CF_3_•, CF_2_CO, C*_n_*F_2*n*_ (*n* = 2 to 3), and C*_n_*F_2*n*+1_• (*n* = 2 to 4)] at 13.0 eV, and (**E**) carbon oxides, C_4_F_9_COF, and emerging hydrolysis products at 14.0 eV (CO at 14.7 eV). The values at 400°C from pyrolysis results are used as the baseline. Background gases (O_2_) and photodissociation products (C_5_F_10_ and •COOH) are shown in (**F**). C_5_F_10_ from pyrolysis and hydrolysis are normalized based on the value at 700°C and 13.0 eV, referring to the same injection amount. The ratio between dry and hydrolysis conditions is also shown in (F).

Temperature dependence curves for identified species during pyrolysis and high-temperature hydrolysis are plotted in Fig. 3, to record species with the same appearance temperatures that would suggest generation via a common pathway. Owing to the unavailability of PI cross-sectional data of fluorocarbon intermediates, an accurate quantification of the concentrations and ratios between each species is not achievable. Nevertheless, we can semiquantify different pathways involving products such as CF_2_, CF_3_•, perfluoroalkenes C*_n_*F_2*n*_ (*n* = 2 to 3), perfluoroalkyl radicals C*_n_*F_2*n*+1_• (*n* = 2 to 4), C_4_F_9_COF, CF_2_CO, CO, and CO_2_, as a function of temperature. Notably, CO_2_ and the decarboxylation products C*_n_*F_2*n*_ were first detected above 750°C, slightly higher than CO, CF_2_CO, C_4_F_9_COF, and C*_n_*F_2*n*+1_• at 700°C. Such observation coincides with earlier calculations (*11*, *42*) that the elimination of CO_2_ shows higher Δ*G* over CO elimination. The concurrence of CF_2_CO, C_4_F_9_COF, and C*_n_*F_2*n*+1_• at 700°C indicates that they are the thermal decomposition products from the CO pathway. We have specifically targeted the formation of CF_2_ carbene and CF_3_• radical due to their significance in understanding the elementary steps. These two C_1_ species are detected only at temperatures above 750° to 800°C, suggesting their formation via secondary fissions from C*_n_*F_2*n*_ and C*_n_*F_2*n*+1_•. Because their appearance temperatures are higher than that of C*_n_*F_2*n*_ and that CF_2_ is not being apparently consumed as the temperature increases, it is more likely that the occurrence of shorter-chain C*_n_*F_2*n*_ is via C─C cleavage from C*_n_*F_2*n*+1_• rather than recombination of CF_2_ carbene. At the highest temperature of 975°C, the temperature dependence curves of C*_n_*F_2*n*+1_, CF_2_CO, and C_4_F_9_COF decline, likely due to the high temperature activating the secondary decomposition or reactions with other species, as well as the degradation of C*_n_*F_2*n*+1_ radicals (*43*). Furthermore, species from nonthermal decomposition pathways have different profiles in the temperature dependence curves. For example, for C_5_F_10_ and •COOH at 13.0 eV identified as the photodissociation fragments of PFHxA, their temperature dependence curves demonstrate the content of remaining parent PFHxA not being pyrolyzed as the temperature increases. We have also plotted O_2_, which is part of the background from the atmosphere and cannot be completely evacuated from the instrument. Its temperature dependence curve irregularly fluctuates at a constant level. N_2_ is present in the atmosphere too, but its IE is relatively high at 15.60 eV (*36*) and thus not ionized and hence not detected.

Water vapor acts as a hydrogen and hydroxyl source for radicals and intermediates of thermal PFAS decomposition and facilitates the formation of HF. In a hazardous waste incinerator, hydrocarbon fuel and >10% water vapor (*26*) are present together with the waste stream. However, because of the operational limitations of our system, only low-pressure water vapor can be fed into the system (as excess water will condense inside the MS and oversaturate the signal). The reduced water vapor experiments, however, still fulfill our aim to examine the emerging species that would assist in investigating the hydrolysis products instead of fully mineralizing all PFHxA reactants. In our experiments, 0.3% water and 0.3% PFHxA diluted in argon carrier gas were introduced into the system. The mass spectrum of PFHxA + H_2_O is presented in Fig. 2E at 14.0 eV and 950°C. A few distinct low-mass peaks were boosted compared to pyrolysis experiments. Ions at *m*/*z* = 46.98 are tentatively assigned as the COF• radical. The IE is measured at 10.25 eV, higher than the reported value of 9.7 eV (*44*). From its PIE curve, the ionization only becomes appreciable above 10.2 eV while the signal from 9.7 to 10.2 eV in our experiments may be too low to detect. We also observed enhanced signal of CF_2_O, with measured IE at 13.0 eV comparable to the reference value of 13.04 eV. The formation of CF_2_O via CF_2_ + O_2_ or OH• pathway was investigated (*22*); however, our current study (under O_2_-starved conditions) shows that there exist alternative pathways in the presence of water vapor. The lower abundances of these intermediates also suggest their low formation efficiencies. Some other low-intensity species include the hydrogenated fluorocarbon radical CF_2_H•, CF_2_OH_2_ (possibly from the addition of water to CF_2_), and C_2_H_2_F_2_O_2_. A comparison between the IE (note that there is some fluctuation of the PIE curve below 11.0 eV due to low signal, but the apparent threshold emerges at 11.0 eV) and that of difluoroacetic acid (CF_2_HCOOH, *m*/*z* = 96.09 and IE = 11.05 eV) confirms its structure. Reminiscent of ketene reacting with water to form acetic acid via 1,1-dihydroxyethene intermediate, which is the enol form of acetic acid, we can assume that CF_2_HCOOH is generated via the hydration of CF_2_═C═O. Meanwhile, we have also performed the pyrolysis of CF_2_HCOOH given that previous studies (*45*) have reported ketene generation via pyrolysis of acetic acid at 740° to 760°C. However, besides CO/CO_2_ and HF elimination, only the elimination of hypofluorous acid (IE = 12.71 eV) (*36*) was observed along with a weak signal of the CHF═C═O counterparts, without CF_2_═C═O being formed even at 900°C (fig. S4).

The introduction of water vapor did not change the formation temperature of each species, indicating that no catalytic effect was observed from the water vapor to the temperature thresholds of decarboxylation and decarbonylation processes (Fig. 3, D to F). We have normalized the intensity of C_5_F_10_ at 700°C and 13.0 eV between the conditions with and without water for the same injection rate of PFHxA vapor and scaled the signal intensity of all other ions, respectively. As shown in Fig. 3F, the ratio remains ~1.0 as the temperature increases with slight fluctuation above 925°C. Compared to pyrolysis reactions, the addition of water vapor enhanced the generations of CF_2_ across the entire temperature range investigated and CF_2_CO up to 900°C. Above 900°C, CF_2_CO and C*_n_*F_2*n*+1_• (*n* = 2 to 4) were apparently consumed by secondary decomposition or reactions with water vapor. Other emerging low-abundance hydrolysis intermediates, COF•, CF_2_O, and CF_2_OH_2_, became substantial only when the temperature was above ~800°C, the same as those of C_1_ species (CF_2_ and CF_3_•), and indicate their origin via secondary reactions between C_1_ species and water vapor (which is also the source of OH•).

### Proposed pyrolysis mechanism

**Fig. 4.**
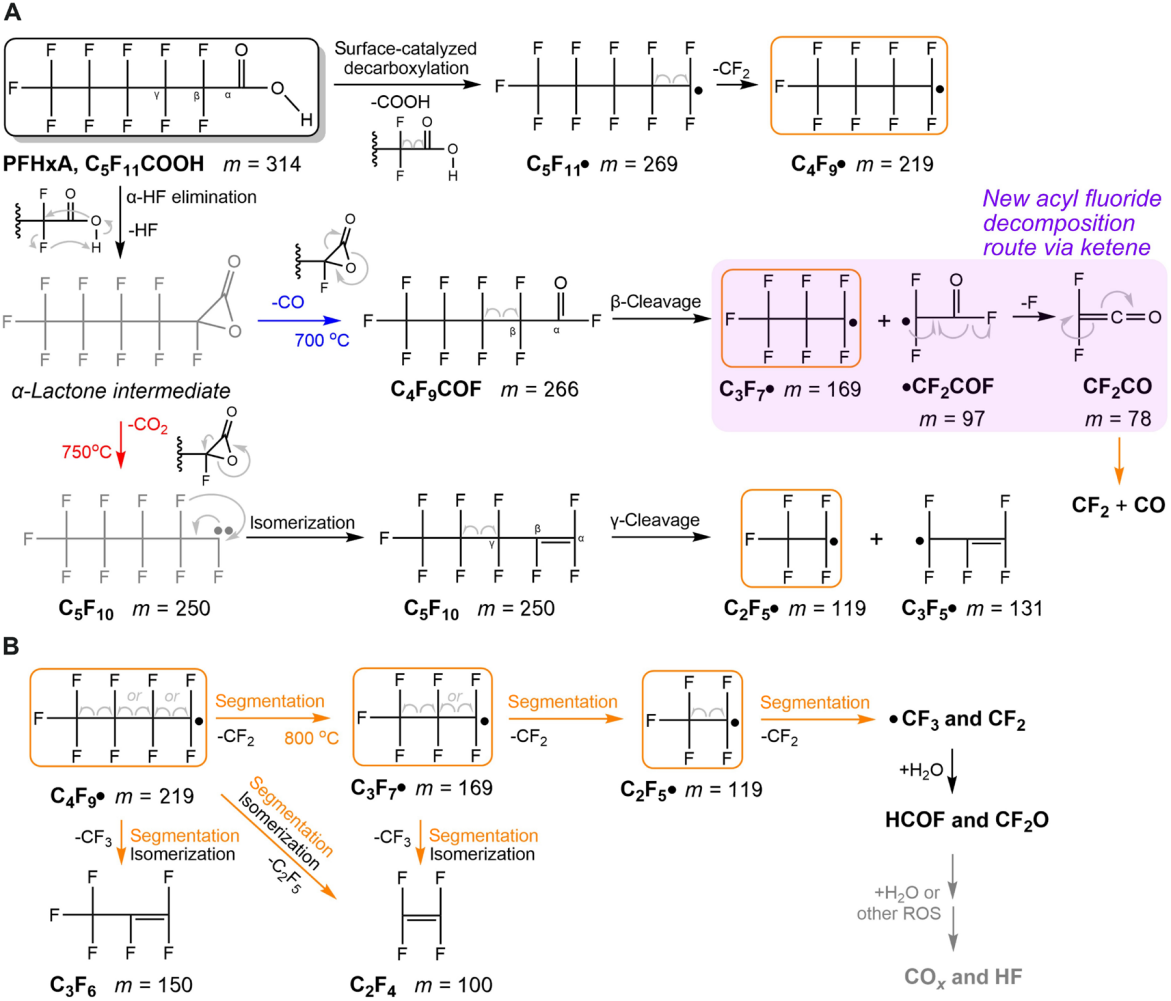
Proposed mechanisms of PFHxA pyrolysis. (**A**) Summary of different thermal decomposition pathways observed in our experiments, including α-HF elimination and subsequent reactions, and surface-catalyzed decarboxylation. The electron-directing arrows are shown in light gray for each step. All structures were experimentally observed except the ones labeled in gray, though implied in the experiments. The decomposition route of acyl fluoride via ketene is highlighted in magenta. Arrows in different colors indicate different temperature thresholds observed. (**B**) The segmentation mechanism of C*_n_*F_2*n*+1_• (*n* = 2 to 4) radicals. The C*_n_*F_2*n*+1_• radicals in the orange frames undergo secondary fission at temperature >800°C to CF_2_ and CF_3_•, which readily react with water or other ROS to become mineralized.

We herein propose the pyrolysis mechanism of PFHxA (Fig. 4) based on our experimental observations and previous calculations (*11*, *21*, *26*, *28*). Our experimental findings align well with the computationally predicted initial HF and CO/CO_2_ elimination pathways (*11*, *25*–*28*). This is mainly due to our low-pressure reaction setup, which reduces secondary reactions and simplifies the reaction network, allowing for a clearer comparison between experimental and theoretical results. PFHxA first eliminates HF at the α-carbon position, which has the lowest barrier among all unimolecular elimination mechanisms, to form an α-lactone intermediate (*11*). Two subsequent reaction pathways, the elimination of CO_2_ and CO, are possible. Higher temperatures (>750°C) initiate the elimination of CO_2_, leading to the product C_5_F_10_ (experimentally indistinguishable from alkene and the carbene form, R_f_-CF:). Successively, shorter C*_n_*F_2*n*_ and/or C*_n_*F_2*n*+1_• fragments were formed via C─C bond cleavage, most likely the C_2_F_5_• moiety loss to C_3_F_5_• (*28*). At temperatures above ~800°C, CF_3_• and CF_2_ were generated from secondary C─C cleavage, validating the proposed mechanisms (*46*–*48*) in thermal decomposition of perfluorocarbons and their radicals. Note that the appearance temperatures of CF_3_• and CF_2_ are higher than that of C*_n_*F_2*n*_, which suggests that the formation of C*_n_*F_2*n*_ is unlikely to result from CF_2_ recombination in our system. The molecular density in our experiments is quite low, and the recombinations of radicals were less likely. As seen from our mass spectra (Fig. 2), no perfluoroalkanes (C*_n_*F_2*n*+2_) were observed, nor were we able to detect 1*H*-perfluoroalkanes via H• or HF rebound to perfluoroalkyl/perfluoroalkene chains.

**Fig. 5.**
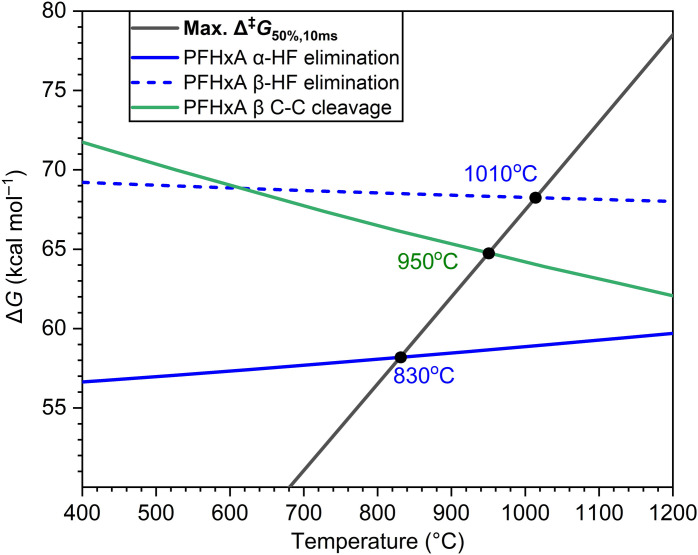
Temperatures for 50% progress (*T*_50_) of each decomposition pathway within 10 ms. Calculated free energies of activation as a function of temperature for α-HF elimination (blue solid line), β-HF elimination (blue dashed line), and β C─C bond cleavage for PFHxA (green solid line). The black line is the maximum free energy of activation required to achieve 50% destruction in 10 ms of residence time (*t*_50_). The temperatures at the crossing points of the respective lowest free energy of activation and the black line represent *T*_50_.

From the α-lactone intermediate, the alternative elimination of CO is favorable, starting from lower temperatures (>700°C), which is consistent with theoretical calculations (*26*, *42*). The acyl fluoride intermediate C_4_F_9_COF was observed. Compared to CO, the signal of C_4_F_9_COF is notably weaker, possibly because of its thermal instability leading to secondary fission reactions. It is worth noting that under gas-phase combustion conditions, the hydrolysis of acyl fluoride into carboxylic acid is ambiguous (*49*), in comparison to the solution phase where this reaction can readily occur via forming tetrahedral intermediates. C_4_F_9_COF will decompose to CF_2_CO and C_3_F_7_• radical via the •CF_2_COF precursor. To confirm that the source of CF_2_CO is acyl fluoride, we conducted a separate experiment on the pyrolysis of perfluorohexanoyl fluoride. The pyrolysis product distribution of perfluorohexanoyl fluoride resembles that of PFHxA, and CF_2_CO and the associated C_4_F_9_• were observed only when the temperature is above 700°C (fig. S5). Such experimental observations confirm that CF_2_CO is indeed a pyrolysis product from acyl fluoride, not a photodissociation fragment. In addition, the elimination of HF and CF_2_CO directly from PFHxA is less likely as the remnant C_3_F_7_COF is absent in the mass spectra. The temperature required for Δ^‡^*G* to achieve 50% of defluorination of •CF_2_COF to CF_2_CO within 10 ms is calculated to be 810°C, which is even lower than that of the initial α-HF elimination of PFHxA at 830°C (Fig. 5). Moreover, high-energy photons may photodissociate •CF_2_COF to CF_2_CO^+^ and F^−^ due to a low calculated energy of only 10.01 eV. These data validate our experimental observation of CF_2_CO as a major decomposition product of C_4_F_9_COF. In addition, the dissociation of CF_2_CO into CF_2_ and CO is favorable, being exothermic by 6.0 kcal/mol (or 0.26 eV) via a dissociation barrier of only 10.0 kcal/mol (or 0.43 eV) (*39*, *50*).

Besides α-HF elimination, there may exist other elimination/dissociation routes. We have calculated and compared the activation free energies (Fig. 5) for α-HF elimination, β-HF elimination, and β C─C bond cleavage for PFHxA using the DLPNO-CCSD(T) method. Decarboxylation (α C─C bond cleavage) was not considered due to a much higher energy barrier compared to the other three pathways (*11*, *26*). The temperature for 50% decomposition via α-HF elimination within 10 ms at 830°C correlates well with half the maximum in temperature dependence curves of nascent products such as C_4_F_9_COF, CF_2_CO, and C*_n_*F_2*n*+1_ (Fig. 3, A and B). This also experimentally validates our computational methodology used in the prediction of the incinerability of PFAS (*11*, *26*). Because β C─C bond cleavage requires a much higher temperature, and the •CF_2_COOH fragment is absent in the mass spectra, β C─C cleavage is considered negligible in our current experiments. Therefore, the formation of C_4_F_9_• is less likely from β C─C cleavage. It is worth mentioning that decarboxylation could occur via surface-catalyzed decomposition, providing one possible source of C_4_F_9_• via thermal decomposition of C_5_F_11_• (observed from the MS background). In previous PFOA pyrolysis experiments (*51*) in an α-alumina reactor at lower temperatures and residence times of about 1 s, it was observed that decarboxylation substantially exceeded decarbonylation, implying that the gas-phase α-lactone route did not predominate. A quantum chemical investigation of this interaction was performed between an Al_6_O_9_ nanocluster and a shorter-chain PFCA (perfluoropropanoic acid). A near-parallel alignment of the PFCA with respect to the Al_6_O_9_ led to the adsorption of an H by an alumina O and the simultaneous adsorption of an F by an adjacent Al atom. This led to decarboxylation and formation of a carbene, which then rearranged into a perfluorinated alkene. If, however, the H of -COOH was directed at an O atom of alumina, but the F chain was not adjacent to the neighboring Al, then the H would be chemisorbed and an O of the -COOH group would then attach to an adjacent Al atom. This will weaken the bond between the -OC═O group and the fluorocarbon chain, facilitating the decarboxylation and releasing a C_*n*-1_F_2*n-*1_ radical. In the case of PFHxA, this radical would be C_5_F_11_•, which undergoes facile scission of a CF_2_ carbene and forms C_4_F_9_•, as shown by kinetic modeling. Other studies of surface catalytic effects of alumina were also reported elsewhere (*52*, *53*).

CF_3_• and CF_2_ readily react with other molecules to form subsequent intermediates depending on the chemical context. In the case of hydrolysis, water acted as a hydrogen source and the precursor for OH• radicals in industrial incinerator environments. In previous studies (*21*, *22*, *54*), reaction pathways were calculated for C_1_ species with H_2_O or OH•. From our experimental observation, direct water addition to CF_2_ leads to the CF_2_OH_2_ intermediate (or CF_2_-H_2_O adduct), quickly decomposing to HCOF (we detected COF• radical instead possibly due to the high temperature–triggered H loss). Alternatively, CF_2_O can be formed via the CF_2_/CF_3_• + OH• pathway. These intermediates further react with water or other reactive oxygen species (ROS), ultimately leading to mineralization. However, fully elucidating these processes would require standalone investigations of CF_2_O/HCOF reactions with water or other ROS, which are beyond the scope of this study. Following this thread, CF_3_• and CF_2_ play a pivotal role in the complete hydrolysis of PFCAs into inorganic fluorine. Therefore, the formation of CF_3_• and CF_2_ induced by higher-temperature environments (>800°C) together with a plethora of hydrogen/hydroxyl sources (such as water vapor and fuel) is necessary. This finding is consistent with the mechanism proposed by Weber *et al.* (*21*, *22*). C─C bond cleavage will gradually become dominant as the temperature further increases. As shown in Fig. 3, the perfluoroalkyl radicals and CF_2_CO intermediates are consumed at higher temperatures, while CO/CO_2_, perfluoroalkenes (C*_n_*F_2*n*_), and CF_2_/CF_3_• continue to accumulate in the system. In addition, we observe a minor accumulation of perfluoroallene (C_3_F_4_) starting from temperatures above 900°C, which we attribute to the defluorination of the C_3_F_5_• radical. Extrapolation of these trends suggests a continued rise of these species with the depletion of other intermediates at operation temperatures below 1200°C inside hazardous waste incinerators (*55*).

On the other hand, the direct elimination of terminal CF_3_• radicals from PFHxA beyond decarboxylation/decarbonylation is not observed. Also, shorter-chain PFCAs and their photodissociation fragments were missing in the spectra, indicating that the hydration of acyl fluorides to shorter-chain PFCAs is not likely to occur under our experimental conditions. Besides, there may also exist other minor pathways like the loss of a carboxyl group and an F• radical, which will not be discussed here due to a lack of experimental evidence.

## DISCUSSION

In the current study, we have provided experimental evidence of fluorocarbon radicals to support and refine the pyrolysis mechanism of PFHxA at the molecular level. The measured initial decomposition temperature aligns with high-level DLPNO-CCSD(T) calculations, showing 50% decomposition via α-HF elimination within 10 ms at 830°C. Successively, we identified radicals and intermediates involved in the conventional decarboxylation (initiated at high temperatures >750°C) and decarbonylation (>700°C) mechanisms. Notably, we observed the exotic difluoroketene intermediate, which has never been reported in previous studies on PFCA pyrolysis. A reaction pathway from acyl fluoride to CF_2_CO and, lastly, CF_2_ and CO was proposed. As the temperature further increases, the segmentation of the carbon backbone becomes dominant. The introduction of water vapor did not affect the appearance temperature threshold for each pathway but enhanced the production of pyrolysis products. Water vapor reacted with CF_2_ carbene and CF_3_ radical to form simple oxygenated fluorocarbons such as CF_2_O and HCOF, which would be further converted into HF, CO, and CO_2_. However, HF was not detected in our system possibly because of the low signal intensity and its reactivity with or absorption/retardation by the alumina reactor. For similar reasons, some extremely reactive species such as F and F_2_ were also not detected.

Unlike traditional pyrolysis experiments performed in furnaces, our SVUV-PIMS setup operates with millisecond-scale residence times and low molecular density, enabling the identification of metastable intermediates. Besides, the injection of water vapor is also limited to prevent signal saturation; thus, our experiments focus on the formation of emerging species from hydrolysis, rather than fully mineralizing PFHxA reactants and pyrolysis products. Our proposed reaction mechanisms primarily involve unimolecular decomposition, which may also apply inside hazardous waste incinerators (*56*). However, it is also worth noting that these incinerators often operate under different conditions compared to our reactor, such as longer residence times (~2 s), higher pressures, and different compositions of the PFAS-containing waste streams. These factors may influence the temperature thresholds for each decomposition pathway and the product distribution by promoting secondary reactions that consume perfluoroalkenes and CF_2_/CF_3_•. Further experiments are required to explore these subsequent reactions.

The identification of key intermediates, mechanisms, and kinetics of pyrolysis is fundamental for optimizing thermal processes. Our work provides a critical understanding of the optimization of incineration conditions to minimize products of incomplete destruction and inform the design of more efficient treatment technologies from a mechanistic perspective, such as the addition of additives or catalysts to enhance PFAS mineralization. Moving forward, our future research will focus on transitioning from laboratory conditions to industrial incineration conditions and exploring radical recombination reactions to improve overall fluorocarbon degradation outcomes.

## MATERIALS AND METHODS

### Experimental design

The experiment was performed at the Combustion and Flame Endstation in the National Synchrotron Radiation Laboratory, Hefei, China (*32*, *57*–*64*). The apparatus (Fig. 1) consists of a pyrolysis chamber with a laminar flow tubular reactor, a differentially pumped molecular beam sampling system, and an SVUV PI chamber mounted with an in-house reflectron time-of-flight mass spectrometer. The flow tube is made of α-alumina (α-Al_2_O_3_) to reduce the surface reactions. It was electrically heated with the heating length of 400 mm and the inner diameter of 7.0 mm to achieve adequately homogeneous reaction conditions. Before the experiments, the sample container was carefully cleaned and dried, and the transfer line (stainless steel) was baked and flushed with argon (Ar, 99.9995%). No fluorocarbon peaks were observed from the background.

### Sample injection

In the experiment, perfluorohexanoic acid (98%, Shanghai Macklin Biochemical Technology Co. Ltd.) was injected into a vaporizer heated to 220°C with the flow rate controlled by an injection pump at 21.88 × 10^−3^ ml/min. Because of the internal flow of cooling Ar gas and heat transfer considerations, the actual sample temperature inside the vaporizer would be lower than this externally measured temperature. The gas vapor was then carried out by inert argon (Ar, 99.9995%) and krypton (Kr, 99.999%) into the transfer line, mixed sufficiently before entering the flow reactor at flow rates of 987 and 10 SCCM (standard cubic centimeters per minute, 273.15 K), respectively, and controlled by mass flow controllers (MKS, Andover, MA, USA). The inlet mole fraction of perfluorohexanoic acid was determined to be 0.3% with a total flow rate of 1000 SCCM (273.15 K). As previous studies reported surface-catalyzed decomposition of PFCAs by GACs (*15*) and quartz (*65*) at temperatures as low as 200°C, our setup differs from previous studies, where our samples are maintained under vacuum and protected by a continuous flow of low-pressure Ar gas, as described above. In addition, Krusic *et al.* (*65*) reported 1*H*-perfluoroheptane and perfluoro-1-heptene as the major products during the decomposition of PFOA at 370°C in a quartz ampoule. However, from our experiments, as seen in Fig. 2C (the photodissociation of PFHxA), none of the 1*H*-perfluoroalkanes or perfluoroalkyl radicals were observed. C_5_F_10_ was indeed observed, but it has a higher appearance energy at ~11.5 eV together with other fragments of PFHxA (Fig. 2A, C_5_F_10_ is missing at 400°C and 11.0 eV). In contrast, the IE of C_5_F_10_ was measured at 10.6 eV as a pyrolysis product in Fig. 2B and fig. S2. On the basis of these observations, we conclude that no evident surface-catalytic decomposition occurred during vaporization and sample injection.

### Data collection

The gas mixture entered an alumina flow reactor heated to between 400° and 975°C and measured every 25°C from 700° to 975°C, monitored by a type S thermocouple. The pressure inside the tube was maintained at 30 torr by an MKS throttle valve (253B-20-40-2, MKS Instruments Inc.) to reduce the molecular collisions so that the metastable intermediates could survive. The products were sampled by a 350-μm quartz nozzle located 10.0 mm downstream of the flow tube outlet, forming a molecular beam into the PI chamber through a 2-mm Ni-skimmer, photoionized by SVUV light (*66*, *67*) and eventually detected by the TOF-MS (*68*). It should be emphasized that the molecular beam sampling coupled with the soft-ionization SVUV-PI technique is capable of probing the intermediates/products in situ without destroying the original chemical structures or the initial chemistry inventory (*57*, *58*). A set of hydrolysis experiments was also performed by just partially replacing inert gas with water vapor, the latter of which was also injected through an injection pump into another vaporizer (130°C) at a flow rate of 2.41 × 10^−3^ ml/min. Therefore, the inlet mole fractions of perfluorohexanoic acid and water vapor were both held at 0.3%. Our measurements inherently involve multiple sampling points, as we collected mass spectra at eight different photon energies within the same reaction temperature. Each of our measurements comprises 3,600,000 to 5,400,000 microscans, providing robust statistical sampling, and thereby enhancing signal quality and reliability. Second, as our results only describe pyrolysis qualitatively, we reference previously established uncertainty ranges of 15 to 20% from published literature (*69*, *70*) for similar measurements. Some other sources of uncertainty may arise from the temperature control, which is within ±5°C. The photon energy from the synchrotron is calibrated and determined within ±0.005 eV uncertainty. A stability test was performed by tracking the signal intensity variance of a specific peak at each temperature, which is within 10%. Other parameters, such as photon flux, chamber pressure, etc., were monitored in real time during experiments to ensure no apparent variation in the experimental conditions.

### Computational methods

Geometries were optimized in Gaussian 16 (*71*) using the ωB97X-D hybrid functional (*72*) with the 6-311+G(2d,2p) basis set (*73*). Single-point energies together with vertical ionization energies for some species were calculated at stationary points using DLPNO-CCSD(T) (*74*, *75*) with the aug-cc-pVTZ basis set (*76*, *77*) in ORCA (*78*–*80*). The maximum Δ^‡^*G* required to achieve 50% destruction within 10 ms (max. Δ^‡^*G*_50%,10ms_) corresponding to the experimental conditions was calculated based on the equation

$${\text{max}.∆}^{‡}G_{50\%,10\text{ms}}=\mathrm{RT}\left[\mathrm{ln\kappa}\left(T\right)+\text{ln}\frac{k_{B}T}{h}-\text{ln}k_{50}\right]$$

where *R* is the universal gas constant, *T* is the absolute temperature, κ is the tunneling correction, *k*_B_ is the Boltzmann constant, *h* is Planck’s constant, and *k*_50_ is the rate constant for 50% destruction within 10 ms, which equals −ln0.5/10 ms.

The determined Δ^‡^*G* values over the temperature range from 1 to 2000 K were then plotted against the max. Δ^‡^*G*_50%,10ms_ of each decomposition pathway. The crossing points of each Δ^‡^*G*(*T*) against the max. Δ^‡^*G*_50%,10ms_ determine the temperatures to attain 50% decomposition within 10 ms. More details can be found in our previous publication (*26*).
