# Supplementary material for: Direct measurement of fluorocarbon radicals in the thermal destruction of perfluorohexanoic acid using photoionization mass spectrometry
Source: Sci Adv. 2025 Feb 28;11(9):eadt3363. doi: 10.1126/sciadv.adt3363 (PMC11870085; doi:10.1126/sciadv.adt3363)
Supplement: Supplementary file 1 — Figs. S1 to S5 Table S1 [file sciadv.adt3363_sm.pdf]

Science Advances
AAAS

Supplementary Materials for

Direct measurement of fluorocarbon radicals in the thermal destruction of perfluorohexanoic acid using photoionization mass spectrometry

Ming-Gao Xu *et al.*

Corresponding author: Long Zhao, zhaolong@ustc.edu.cn; Wenchao Lu, wenchao.lu@csiro.au

Sci. Adv. **11**, eadt3363 (2025)
DOI: 10.1126/sciadv.adt3363

This PDF file includes:

Figs. S1 to S5
Table S1

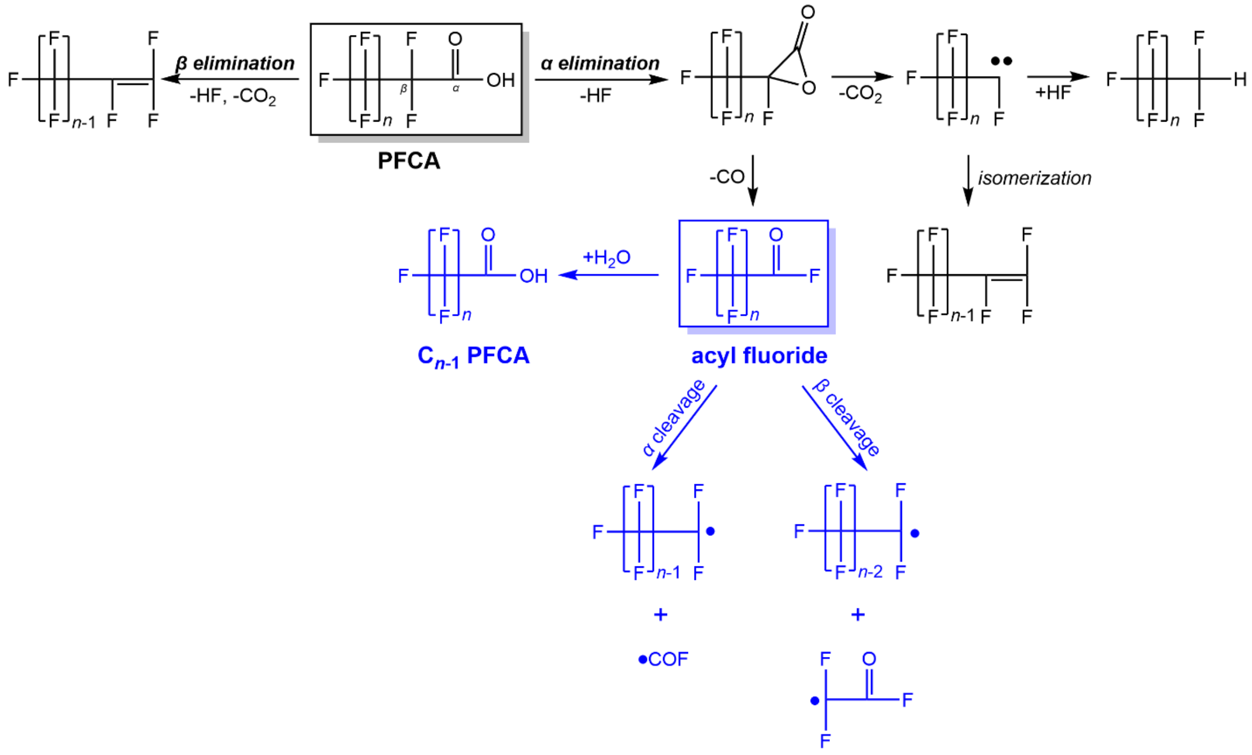

Fig. S1.

Previously proposed thermal decomposition mechanisms of perfluorocarboxylic acids (PFCAs), based on References 11 and 25 – 28. A key intermediate, perfluorinated acyl fluoride, and its subsequent degradation pathways are colored in blue. Surface-catalyzed reactions such as those on granular activated carbons (GACs) are not shown.

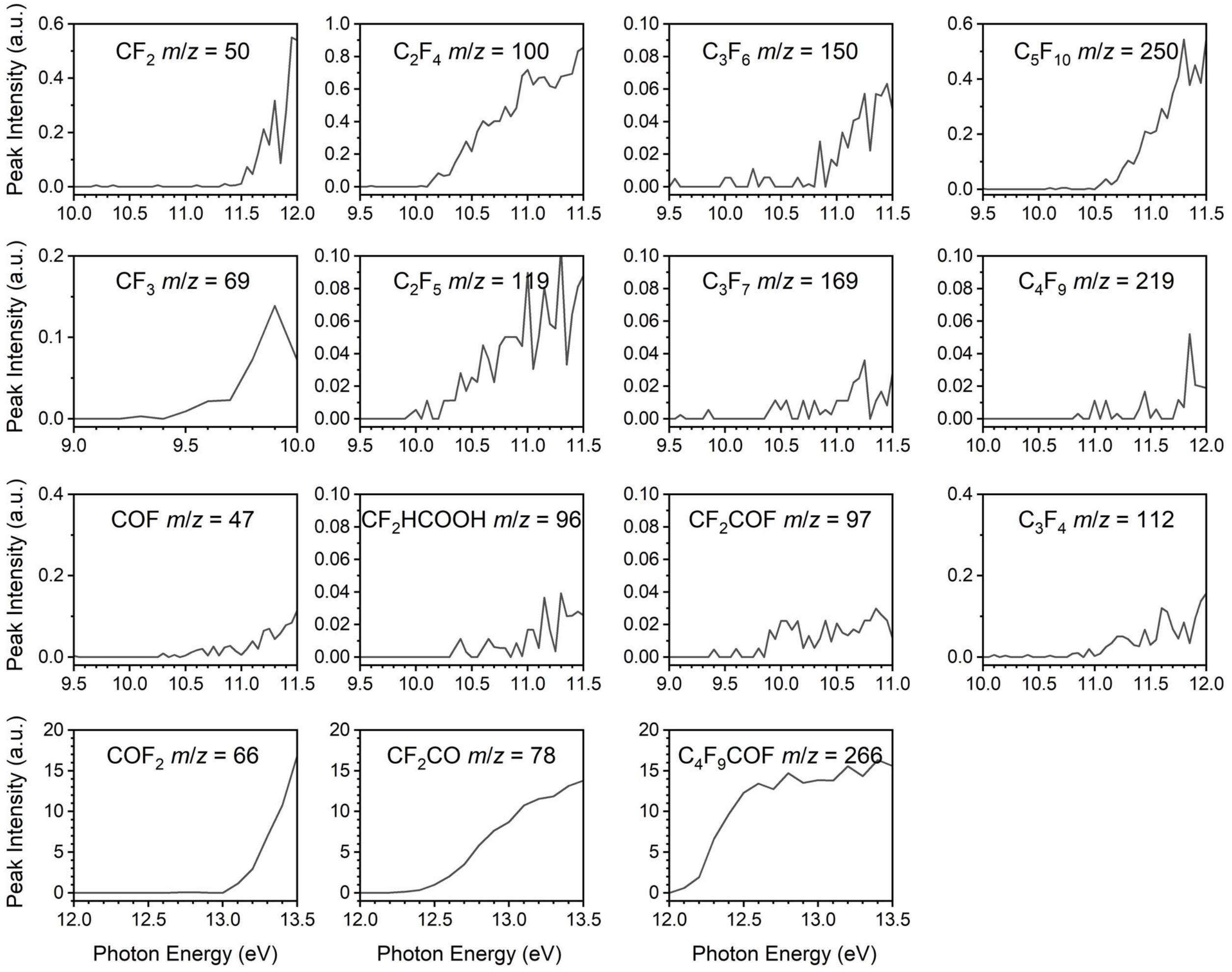

Fig. S2.

Ionization threshold measurements and photoionization efficiency curves for all thermal products from the pyrolysis of PFHxA in this study.

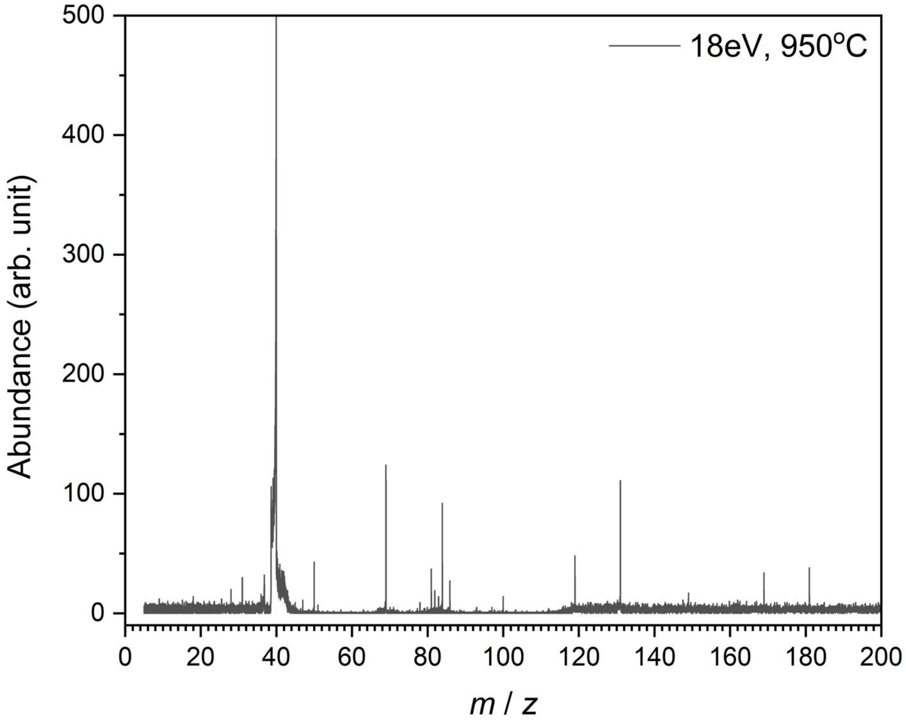

Fig. S3.

MS collected for PFHxA at 18.0 eV and 950 °C, indicating the absence of HF, F, and F_2. The peak of nominal mass 40 is argon (*m*/*z* = 39.962).

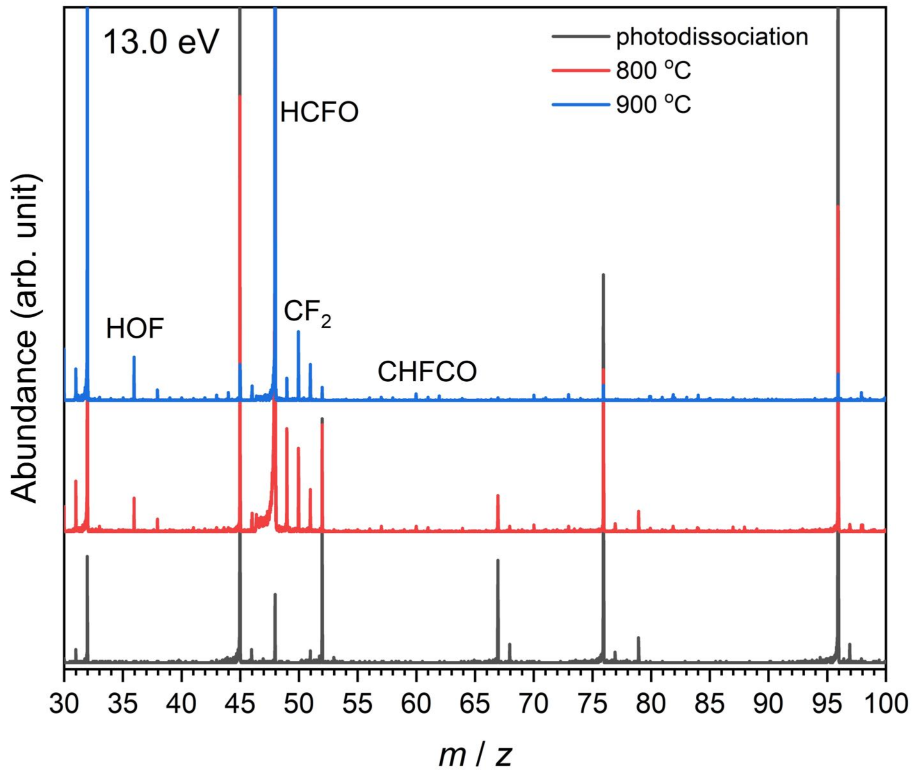

Fig. S4.

Pyrolysis products of difluoroacetic acid, CF_2HCOOH (*m*/*z* = 96), recorded at 13.0 eV and various temperatures, indicating no water elimination product CF_2=C=O observed.

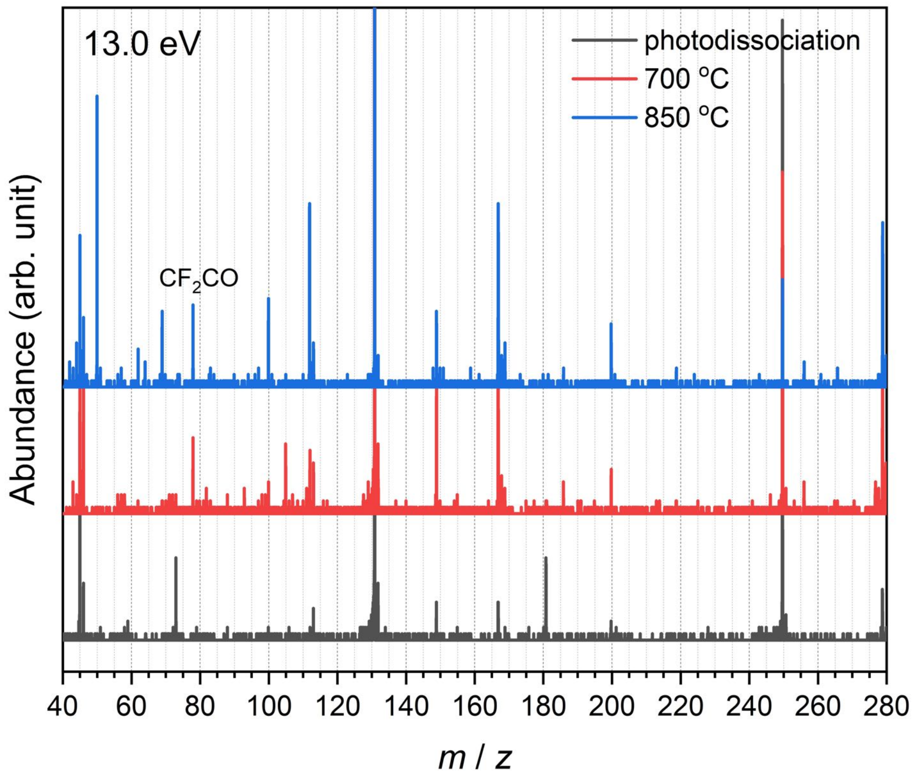

Fig. S5.

Pyrolysis products of perfluorohexanoyl fluoride, $C_5F_{11}COF$ (*m*/*z* = 316), recorded at 13.0 eV and various temperatures, highlighting the formation of CF_2=C=O via pyrolysis.

Table S1.

Identification of pyrolysis products of PFHxA. The round dot "•" after each formula indicates a radical species, while the others represent closed-shell singlet species.

m/*z*	Formula	CAS #	Appearance Temperature (°C)	Photoionization energy (eV)	
				Exp	Ref [a]
27.99	CO	630-08-0	700	~ 14.0	14.00
43.98	CO_2	124-38-9	750	> 13.5	13.78
46.98	COF•	1871-24-5	800	10.25	9.7
49.99	CF_2	2154-59-8	800	11.4	11.45
65.98	CF_2O	353-50-4	850	13.0	13.04
68.06	CF_2OH_2		Signal too weak	Signal too weak	
68.99	CF_3•	2264-21-3	750 – 800	9.3	9.25
77.99	CF_2CO	683-54-5	700	12.2	
96.09	CF_2HCOOH	381-73-7	Signal too weak	11.0	11.05 [b]
96.99	CF_2COF•		700 – 800	9.9	9.94 [c]
99.99	C_2F_4	116-14-3	750 – 800	10.1	10.12
111.98	C_3F_4	461-68-7	800 – 900	10.8 [d]	10.88
118.98	C_2F_5•	3369-48-0	750	9.9	9.98
149.98	C_3F_6	116-15-4	Signal too weak	10.7	10.60
168.96	C_3F_7•	3170-79-4	700	10.5	
218.96	C_4F_9•	4520-67-6	750	11.4	
245.95	C_5F_{10}	376-87-4	750 [e]	10.6	
265.97	C_4F_9COF	375-62-2	700	12.1	
Other species:					
17.00	OH•	3352-57-6	–	Not detected	13.01
18.00	H_2O	7732-18-5	–	From background	12.62
19.00	F•	14762-94-8	–	Not detected	17.42
20.00	HF	7664-39-3	–	Not detected	16.00
28.00	N_2	7727-37-9	–	From background	15.60
31.98	O_2	7782-44-7	–	From background	12.07
36.00	HOF	14034-79-8	–	Not detected	12.71
38.00	F_2	7782-41-4	–	Not detected	15.69
39.96	Ar	7440-37-1	–	Carrier gas	15.76
313.98	PFHxA	307-24-4	Reactant	Not detected	

[a] From *NIST Chemistry WebBook, NIST Standard Reference Database Number 69.*
[b] Measured values of pure substance
[c] From DLPNO-CCSD(T) calculation
[d] Perfluoroallene, $CF_2{=}C{=}CF_2$
[e] Measured at 11.0 eV. Also observed as a photodissociation fragment from parent $PFHxA^+$ above 11.5 eV
